# Impact of deep learning image reconstruction on ADC quantification and histogram metrics: a phantom study

**DOI:** 10.1186/s41747-026-00709-y

**Published:** 2026-04-13

**Authors:** Simona Marzi, Vicente Bruzzaniti, Francesca Laganaro, Giovanni Di Giulio, Michele Farella, Alessia Tonnetti, Irene Terrenato, Roberto Castellana, Antonello Vidiri

**Affiliations:** 1 Medical Physics Laboratory, IRCCS Regina Elena National Cancer Institute, Rome, Italy; 2 Radiology and Diagnostic Imaging Department, IRCCS Regina Elena National Cancer Institute, Rome, Italy; 3 Department of Clinical Engineering and Information Technology, IRCCS Regina Elena National Cancer Institute, Rome, Italy; 4 Biostatistics and Bioinformatics Unit, IRCCS Regina Elena National Cancer Institute, Rome, Italy

**Keywords:** Artificial intelligence, Deep learning, Diffusion magnetic resonance imaging, Image processing (computer-assisted), Radiomics

## Abstract

**Objective:**

Recently, deep learning (DL)-based reconstruction methods have been introduced into clinical magnetic resonance imaging (MRI) systems to enhance image quality and reduce acquisition time. However, their effects on apparent diffusion coefficient (ADC) maps remain unclear. We investigated whether DL-based image reconstruction influences ADC quantification and histogram-based ADC metrics using a calibrated diffusion-weighted imaging (DWI) phantom.

**Materials and methods:**

A phantom containing vials with known ADC values was scanned on a 3-T system using full (fFOV) and reduced (rFOV) field-of-view DWI sequences. Each acquisition was performed using conventional (DL-OFF) and three DL-based strength levels (low, medium, high). Median ADC values were analyzed for repeatability (coefficient of variation (CV)) and accuracy. Histogram changes and first-order radiomic features were assessed using the Wasserstein distance, Friedman, and Wilcoxon tests.

**Results:**

ADC estimates showed high repeatability (CV 0.1–1.2%) and good accuracy (deviation -2 to 7%) across all DL levels and sequences. DL reconstruction progressively reduced histogram dispersion, particularly in high-ADC vials. Wasserstein distances increased with DL strength, confirming a progressive effect on ADC value distributions, while median ADC values remained unchanged. Entropy and interquartile range decreased significantly (*p* < 0.001), whereas kurtosis and skewness increased, with differences showing less stable and sequence-dependent statistical significance.

**Conclusion:**

DL-based reconstruction maintained accurate and repeatable ADC quantification while reducing the dispersion of ADC values. The effect was more evident for high-ADC regions and the rFOV sequence, resulting in narrower distributions of ADC values. Further investigations comparing different DL-based solutions are warranted to assess the generalizability of these findings in clinical settings.

**Relevance statement:**

Over the past decade, ADC histogram analysis has proven valuable for quantifying tumor heterogeneity, differentiating tumor grade, and evaluating early treatment response. Deep learning reconstruction narrows ADC distributions and reduces dispersion, supporting its potential in oncologic DWI, while highlighting the need for patient-based validation studies.

**Key Points:**

- DL reconstruction preserved ADC accuracy in both full FOV and reduced FOV DWI.
- ADC repeatability remained high across DL levels for both DWI sequences.
- Histogram dispersion progressively reduced across DL levels, particularly in high-ADC vials.
- Entropy and interquartile ranges decreased progressively with increasing DL strength.

**Graphical Abstract:**

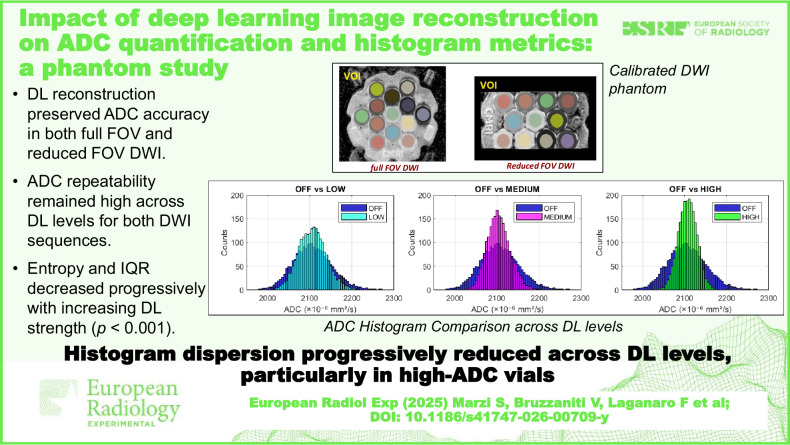

## Objective

Diffusion-weighted imaging (DWI) is routinely used in clinical magnetic resonance imaging (MRI), providing quantitative information on tissue microstructure and water diffusivity. The apparent diffusion coefficient (ADC) is widely recognized as a non-invasive quantitative imaging biomarker for tissue characterization in oncology, reflecting cell density and microstructural barriers to diffusion [1, 2].

Histogram analysis of ADC maps allows the assessment of the full distribution of water diffusivity values within a lesion, providing complementary information beyond the mean or median ADC [3]. Also known as first-order radiomics, this approach has gained widespread use over the past decade due to its simplicity and the robustness of derived features. Parameters such as kurtosis, skewness, entropy, and percentiles have been shown to reflect tumor heterogeneity, microstructural complexity, and treatment-related changes across several tumor types [4–13]. However, these histogram-derived features are intrinsically sensitive to signal-to-noise ratio (SNR), artifacts, and voxel-level variability, which may affect their quantitative reliability.

Recently, deep learning (DL)-based reconstruction methods have been introduced into clinical MRI systems to enhance SNR and reduce acquisition time [14–16]. While these methods improve image appearance, their effects on quantitative diffusion parameters and ADC maps are unclear. In particular, DL-induced denoising could modify the shape of ADC histograms by reducing noise-driven variability and potentially smoothing heterogeneity.

To ensure reliable diffusion quantification, standardized phantoms with known ADC values are essential [17–20]. These calibration tools, developed through international initiatives such as the Quantitative Imaging Biomarkers Alliance (QIBA) [17, 19], provide reference measurements that are independent of the scanner and protocol, enabling robust assessment of ADC accuracy and repeatability under controlled conditions. Such controlled scenarios allow isolation of reconstruction-related effects that are difficult to disentangle *in vivo* due to biological variability and motion.

The purpose of this study is to evaluate the impact of different DL reconstruction strengths on ADC quantification and histogram using a National Institute of Standards and Technology (NIST)/QIBA-calibrated diffusion phantom. We hypothesized that increasing DL reconstruction strength could systematically influence ADC value distributions and first-order histogram features.

To capture potential effects related to spatial resolution, both a standard full-field-of-view (fFOV) and a reduced-field-of-view (rFOV) DWI sequence were analyzed, the latter commonly used to evaluate subcentimeter-sized anatomical structures or lesions [21]. This analysis aims to clarify the extent to which DL reconstruction preserves the quantitative integrity of ADC measurements and their histogram-based metrics using two DWI sequences with different spatial resolutions, providing a more comprehensive assessment of DL-related effects.

## Materials and methods

### DWI acquisition protocols

DWI was performed on a 3-T system (Signa™ Premier, GE Healthcare) using a dedicated 21-channel receive-only head and neck radiofrequency coil. Two DWI sequences were implemented: full field-of-view (fFOV) DWI and reduced field-of-view (rFOV) DWI. Both sequences used single-shot echo-planar imaging, with the rFOV sequence employing a two-dimensional spatially selective excitation to reduce the excited volume. Five *b*-values (0, 500, 1,000, 1,500, and 2,000 s/mm²) were applied in both sequences, with diffusion-sensitizing gradients along three orthogonal directions to obtain trace-weighted images. Two averages were acquired at *b* = 0 s/mm² and four averages at *b* = 500, 1,000, 1,500, and 2,000 s/mm² for rFOV DWI, whereas two averages were acquired for each *b*-value in fFOV DWI. A higher number of averages was used for rFOV DWI to compensate for its intrinsically lower signal-to-noise ratio (SNR), resulting from the reduced excited volume and higher spatial resolution [22]. Imaging parameters are detailed in Table 1.

**Table 1:** DWI acquisition parameters

Parameters	fFOV DWI	rFOV DWI
TR/TE (ms)	8,000/65.9	4,500/60.6
FOV (mm)	220	180
Acquisition matrix	128 × 128	100 × 50
Reconstruction matrix	256 × 256	256 × 256
Slice thickness (mm)	4	3
Pixel size (mm^2^)	0.86 × 0.86	0.70 × 0.70
No. of slices	25	27
*b* values (s/mm^2^)	0, 500, 1,000, 1,500, 2,000	0, 500, 1,000, 1,500, 2,000
Acceleration factor	2	None
Acquisition time	3:40 min	3:50 min
Flip angle	90	90
Bandwidth (Hz/Px)	1,953.1	1,953.1
Acquisition type	2D	2D

### Phantom experiments

Our 3-T MRI system was equipped with a DL-based reconstruction software (AIR™ Recon DL, GE Healthcare) designed to improve SNR and enhance image sharpness. This vendor-provided DL reconstruction model was based on a deep convolutional neural network trained in a supervised learning scheme on a large dataset of paired conventional and near-perfect MR images [23]. Four reconstruction methods were applied for both DWI sequences: conventional reconstruction (DL off) and DL-based reconstruction with low, medium, and high strength levels. Each sequence was repeated five times within the same measurement session to assess short-term reproducibility, and a second session was performed 1 month later to evaluate long-term reproducibility.

**Fig. 1:**
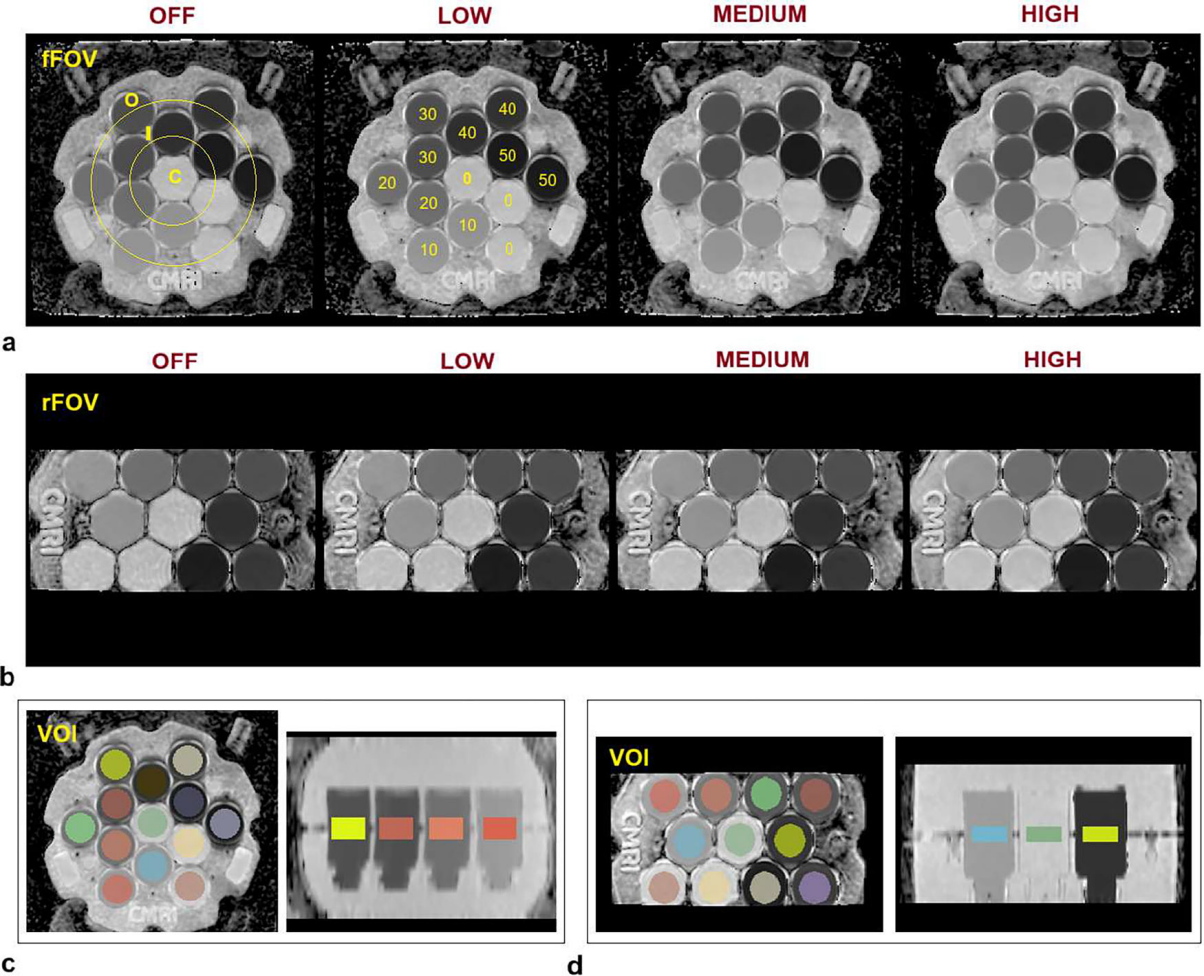
ADC maps acquired at increasing deep learning levels using (**a**) full field-of-view (fFOV) and (**b**) reduced field-of-view (rFOV) DWI sequences. The phantom structure and vial arrangement are shown in **a**, where the central (C), inner (I), and outer (O) vial positions and the corresponding PVP concentrations (0–50%) are indicated. The volumes of interest (VOIs) used for evaluating ADC distributions are shown overlaid on (**c**) axial and sagittal views for the fFOV maps, and on (**d**) axial and coronal views for the rFOV maps

All acquisitions were performed using a commercial diffusion phantom (NIST/QIBA Diffusion Phantom, CaliberMRI, Boulder, CO, USA) [24, 25]. The phantom contains thirteen 20 mL vials with six different ADC values ranging from 0.3 × 10⁻³ to 2.0 × 10⁻³ mm²/s at 20 °C, obtained from polyvinylpyrrolidone (PVP) solutions of six concentrations (0%, 10%, 20%, 30%, 40%, and 50% by weight). Vials with identical PVP concentrations are positioned at different locations within the phantom, arranged in the middle, inner, and outer rings, as shown in Fig. 1. Because the rFOV sequence covers a smaller field of view, the phantom was rotated by 90° during acquisitions to include as many inserts as possible, *i.e*., 11 out of 13 (Fig. 1). The same DL reconstructions were applied identically across all acquisitions and independently of phantom orientation within the FOV.

The dependence of ADC on temperature for each PVP concentration was characterized by the National Institute of Standards and Technology (NIST) in the 15–24 °C range at 3.0 T and supplied with the phantom documentation. The phantom, which includes an integrated liquid-crystal thermometer (precision ± 1 °C), was left in the magnet room overnight before each measurement session to ensure thermal equilibrium. A dedicated 3D T1-weighted spoiled gradient-echo sequence was acquired before and after each measurement session to estimate the internal phantom temperature, as recommended by the manufacturer’s user manual.

Nominal ADC values for each PVP concentration at the measured temperature were calculated using a second-order polynomial model with the Curve Fitting Toolbox (cftool) in MATLAB (Release 2021b).

### ADC histogram analysis

ADC maps were generated using a commercial software package (FuncTool application, version 4.4, GE Healthcare).

The 3D Slicer software [26] was used to visualize the ADC maps and delineate the volumes of interest (VOIs). Each VOI was defined as a cylindrical region obtained by drawing a 2.5 cm− or 2.2 cm-diameter circle on three consecutive slices and placing it at the center of each vial, for fFOV and rFOV DWI, respectively. VOI delineation was performed in a semi-automatic manner, with circular regions of interest manually positioned and then replicated across slices to form cylindrical VOIs. The slightly smaller diameter used for rFOV images was chosen to minimize potential edge effects resulting from the reduced field of view, as illustrated in Fig. 1. The resulting contours were exported as binary masks and imported into MATLAB for quantitative analysis.

The same VOIs were applied identically across all reconstruction conditions (DL OFF, LOW, MEDIUM, and HIGH) to avoid segmentation-related variability.

ADC values from all voxels within each VOI were aggregated to obtain the corresponding ADC distribution. Histograms were then computed using a fixed bin size of 5 × 10⁻⁶ mm²/s. A predefined ADC vector (Xvector), ranging from the minimum to the maximum ADC values and sampled at this fixed bin, was used to define the histogram bins. Mean histograms for each DL level were obtained by averaging the bin counts across the five repeated measurements. The same Xvector was applied across all DL levels to ensure consistent binning.

Eleven first-order radiomic parameters were then extracted from the ADC histograms within each VOI, including mean, standard deviation, median, interquartile range (IQR), 10th, 25th, 75th, and 90th percentiles, kurtosis, skewness, and entropy. These metrics were used for subsequent statistical analyses.

### Statistics

Statistical analyses were performed using MATLAB (Release 2021b, MathWorks) and IBM SPSS (version 30.0). Statistical significance was set at *p* < 0.05.

Statistical analyses were organized to evaluate: (1) ADC accuracy relative to nominal reference values, (2) ADC short-term repeatability (measured by intra-session coefficient of variation and maximum percentage deviation), (3) differences in ADC histograms between reconstructions using the Wasserstein distance, and (4) group-wise comparisons of ADC histograms and first-order radiomic features across DL reconstruction strengths using the Friedman test and paired Wilcoxon signed-rank tests, respectively.

Agreement between measured and nominal ADC values was assessed by calculating the percentage deviation between these values ((measured - nominal)/nominal × 100). Median ADC values within each VOI were used for this analysis, as the distributions did not meet normality according to the Shapiro–Wilk test. These values were then averaged across the five repeated acquisitions within each session.

Short-term repeatability was evaluated using the intra-session coefficient of variation (CV = 100 × standard deviation/mean, expressed as %). To further assess short-term measurement stability, the maximum percentage deviation between the first and subsequent repeated measurements was calculated for each vial and DL reconstruction level.

To capture potential differences in the entire ADC value distribution, the Wasserstein distance was computed between the distributions obtained with conventional reconstruction (DL OFF) and those from each DL reconstruction strength level [27]. Specifically, for each pair of ADC histograms, cumulative frequency curves were derived by summing the relative bin counts across the ADC range. The Wasserstein distance was then obtained as the total absolute difference between these cumulative curves. This metric provided an interpretable measure of the “distance” required to transform one distribution into another. Wasserstein distances were calculated for each VOI and compared across DL reconstruction strengths and sequence types.

The effects of DL reconstruction strength on the entire ADC value distributions were tested for each VOI and sequence type, using the Friedman test for non-parametric paired data, which enabled simultaneous multiple comparisons across all DL reconstruction levels (DL OFF, LOW, MEDIUM, HIGH).

Differences in first-order radiomic ADC parameters between the DL OFF condition and the various DL levels were evaluated using the Wilcoxon signed-rank test for paired data, applied separately for each sequence and measurement session.

Raw ADC measurements were analyzed separately for each session to avoid temperature-related normalization between acquisitions. Quantitative metrics were then summarized across sessions using metric-specific aggregation strategies, as specified below: coefficients of variation and Wasserstein distances were averaged, maximum deviations were summarized by retaining the extrema, and accuracy bias and percent differences in first-order radiomic features were aggregated by combining session-level deviations. Session-specific results were also reported to assess temporal consistency.

## Results

### ADC accuracy and repeatability

Measurements were performed at 22.9 °C and 20.8 °C for the fFOV sessions, and at 20.8 °C and 22.2 °C for the rFOV sessions (precision ± 1 °C). Estimated fit coefficients and 95% confidence intervals for the nominal ADC-temperature dependence at each PVP concentration are reported in Supplementary Table S1.

For fFOV, deviations of measured ADC values from the nominal values across all inserts and DL reconstruction strengths ranged from -2.1% to 7.3%, while for rFOV, they ranged from 0.8% to 7.2% (Table 2). The magnitude of ADC bias did not show a dependence on DL reconstruction strength. Session-specific results are detailed in Supplementary Fig. S1, indicating good overall accuracy for each measurement session.

**Table 2:** Median and interquartile range of ADC deviations (%) from the nominal references for each vial and sequence, pooled across sessions

	fFOV							
insert	OFF		LOW		MEDIUM		HIGH	
	Median	IQR	Median	IQR	Median	IQR	Median	IQR
PVP0_C	2.6	(4.5)	2.2	(4.0)	2.3	(4.2)	2.3	(4.2)
PVP0_I	5.6	(4.1)	5.2	(4.0)	5.4	(4.0)	5.4	(3.9)
PVP0_O	6.7	(5.4)	6.5	(5.1)	6.5	(5.1)	6.6	(4.7)
PVP10_I	5.0	(5.0)	4.9	(4.9)	4.9	(4.9)	4.9	(4.9)
PVP10_O	7.2	(5.2)	7.2	(5.2)	7.3	(5.4)	7.3	(5.4)
PVP20_I	3.0	(5.1)	3.0	(5.1)	3.0	(5.0)	3.0	(4.9)
PVP20_O	4.9	(4.7)	5.0	(4.5)	5.1	(4.7)	5.0	(4.7)
PVP30_I	1.7	(5.1)	1.6	(5.1)	1.7	(5.0)	1.7	(5.0)
PVP30_O	2.1	(5.0)	2.4	(4.9)	2.4	(4.9)	2.5	(5.0)
PVP40_I	1.9	(5.0)	1.8	(5.2)	1.9	(5.0)	2.0	(5.4)
PVP40_O	4.7	(4.5)	5.0	(4.0)	5.0	(4.0)	5.1	(4.0)
PVP50_I	-2.1	(6.9)	-2.0	(7.2)	-2.0	(6.9)	-2.1	(7.5)
PVP50_O	-1.2	(7.0)	-1.1	(7.0)	-1.1	(7.0)	-0.9	(7.6)

	rFOV							
insert	OFF		LOW		MEDIUM		HIGH	
	Median	IQR	Median	IQR	Median	IQR	Median	IQR
PVP0_C	2.3	(0.3)	2.2	(1.0)	2.1	(0.9)	2.2	(0.8)
PVP0_I	4.0	(0.7)	4.0	(1.1)	3.9	(0.9)	4.0	(0.8)
PVP0_O	5.8	(0.9)	5.6	(1.2)	5.4	(1.5)	6.0	(1.3)
PVP10_I	4.4	(0.5)	4.4	(0.6)	4.3	(0.9)	4.3	(0.8)
PVP10_O	7.2	(0.6)	7.2	(1.1)	7.2	(1.2)	7.0	(0.7)
PVP20_I	4.0	(0.7)	4.3	(1.0)	4.0	(1.1)	4.0	(1.1)
PVP30_I	2.1	(0.7)	2.3	(0.9)	2.1	(0.9)	2.0	(0.9)
PVP30_O	2.4	(1.0)	2.4	(1.2)	2.3	(1.3)	2.1	(1.0)
PVP40_I	1.0	(0.2)	1.0	(0.6)	0.8	(0.6)	0.8	(0.4)
PVP40_O	3.0	(0.5)	2.9	(0.6)	3.0	(0.5)	2.9	(0.5)
PVP50_I	2.1	(0.5)	2.2	(0.8)	2.1	(0.9)	1.9	(0.8)

PVP solutions of six concentrations (0%, 10%, 20%, 30%, 40%, and 50% by weight), arranged in the middle (C), inner (I), and outer (O) rings

*IQR* Interquartile range, *PVP* Polyvinylpyrrolidone

CV values averaged across the two measurement sessions are reported in Table 3; for fFOV, they ranged from 0.13% to 0.43%, while for rFOV, from 0.16% to 0.77%. Session-specific CV values are reported in Supplementary Table S2 and indicate high repeatability of the ADC estimates across all DL reconstruction levels and acquisition sessions.

**Table 3:** Coefficient of variation (CV) across DL reconstruction levels and inserts for both fFOV and rFOV acquisitions, averaged over the two repeated sessions, expressed as %

		fFOV		
insert	OFF	LOW	MEDIUM	HIGH
PVP0_C	0.40	0.37	0.34	0.42
PVP0_I	0.21	0.16	0.17	0.27
PVP0_O	0.42	0.35	0.35	0.43
PVP10_I	0.15	0.14	0.15	0.19
PVP10_O	0.19	0.19	0.20	0.16
PVP20_I	0.19	0.19	0.18	0.21
PVP20_O	0.19	0.19	0.20	0.16
PVP30_I	0.14	0.15	0.17	0.13
PVP30_O	0.15	0.15	0.16	0.15
PVP40_I	0.18	0.18	0.20	0.15
PVP40_O	0.31	0.25	0.27	0.30
PVP50_I	0.24	0.18	0.32	0.23
PVP50_O	0.32	0.18	0.22	0.34

		rFOV		
insert	OFF	LOW	MEDIUM	HIGH
PVP0_C	0.30	0.35	0.32	0.26
PVP0_I	0.30	0.42	0.41	0.26
PVP0_O	0.33	0.54	0.51	0.39
PVP10_I	0.27	0.25	0.25	0.25
PVP10_O	0.43	0.56	0.43	0.35
PVP20_I	0.26	0.32	0.29	0.24
PVP30_I	0.29	0.31	0.33	0.22
PVP30_O	0.34	0.42	0.34	0.26
PVP40_I	0.26	0.45	0.39	0.16
PVP40_O	0.49	0.77	0.73	0.24
PVP50_I	0.31	0.56	0.62	0.40

Polyvinylpyrrolidone (PVP) solutions of six concentrations (0%, 10%, 20%, 30%, 40%, and 50% by weight), arranged in the middle (C), inner (I), and outer (O) rings

Pooled maximum deviations ranged from -1.2% to +1.7% for fFOV DWI and from -2.9% to +1.8% for rFOV DWI. Session-specific maximum percentage deviations are shown in Supplementary Fig. S2 and indicate no evident dependence on the DL level or acquisition session for both fFOV and rFOV sequences.

### ADC histogram analysis

**Fig. 2:**
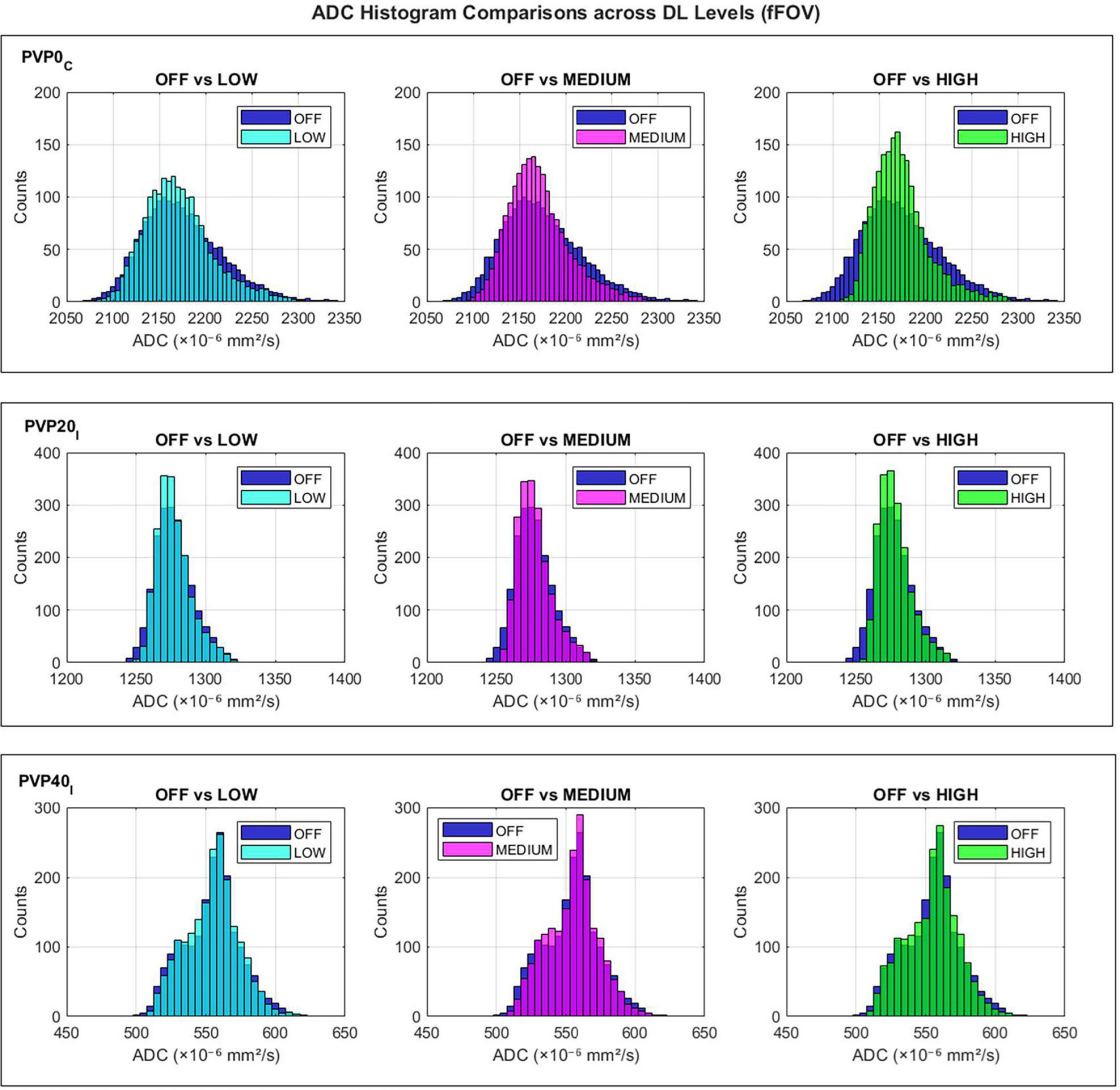
ADC histograms from full FOV (fFOV) DWI acquisitions at increasing DL reconstruction levels. Each row shows a representative vial (PVP0C, PVP20I, PVP40I), with OFF–DL comparisons (LOW, MEDIUM, HIGH) in each column. Mean histograms were calculated by averaging over five repeated measurements from the first measurement session

**Fig. 3:**
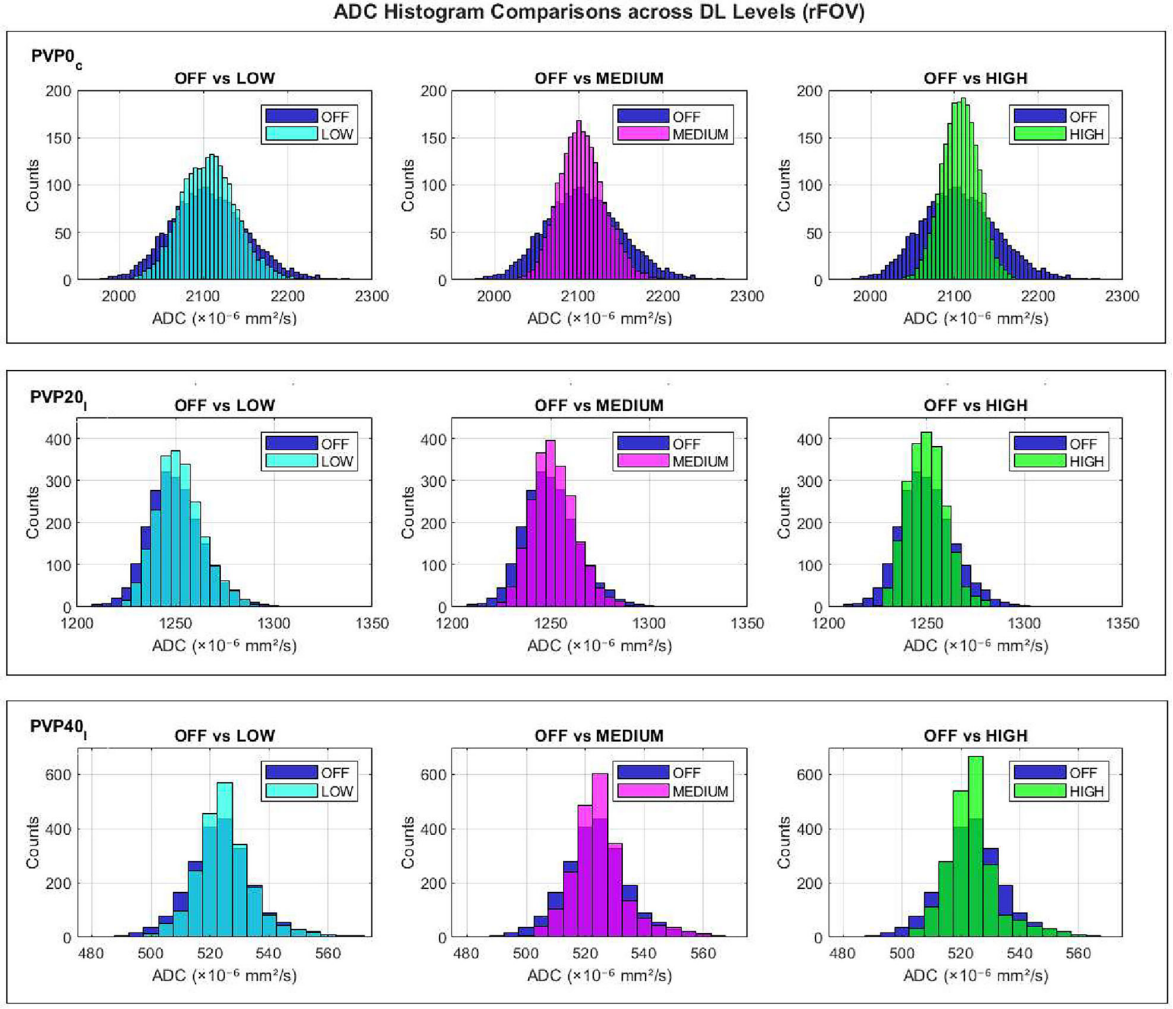
ADC histograms from reduced FOV (rFOV) DWI acquisitions at increasing DL reconstruction levels. Each row shows a representative vial (PVP0C, PVP20I, PVP40I), with OFF–DL comparisons (LOW, MEDIUM, HIGH) in each column. Mean histograms were calculated by averaging over five repeated measurements from the first measurement session

A comparison of ADC histograms from the first measurement session of fFOV and rFOV DWI at increasing DL reconstruction levels is shown for three representative inserts (PVP0C, PVP20I, and PVP40I) in Figs. 2 and 3, respectively. Comparable histogram patterns were obtained for the second session. With a stronger DL level, the histograms became progressively sharper, particularly for the high-ADC vial (PVP0C). Less pronounced effects were observed for intermediate (PVP20I) and low-ADC (PVP40I) vials.

Session-averaged OFF–HIGH Wasserstein distances were highest for the water vials, ranging from 8.3 to 11.8 in fFOV and from 21.1 to 21.7 in rFOV DWI, whereas all other inserts showed substantially smaller distances (≤ 3.1 and ≤ 9.2, respectively). OFF–LOW and OFF–MEDIUM reconstructions exhibited intermediate values. More details are presented in Supplementary Fig. S3 and Supplementary Table S3.

Friedman test results for differences in ADC distributions across DL levels, for each insert and session, are summarized in Supplementary Table S4. Statistically significant differences were observed for all water vials in both sequence types and both sessions (all *p* < 0.001). In fFOV acquisitions, only 3 of 10 non-water inserts showed significant differences in both sessions, whereas in rFOV acquisitions, 8 of 11 inserts were significantly different across both sessions, in agreement with the larger Wasserstein distances observed for this sequence.

### Effect of DL strength on first-order radiomic ADC parameters

**Fig. 4:**
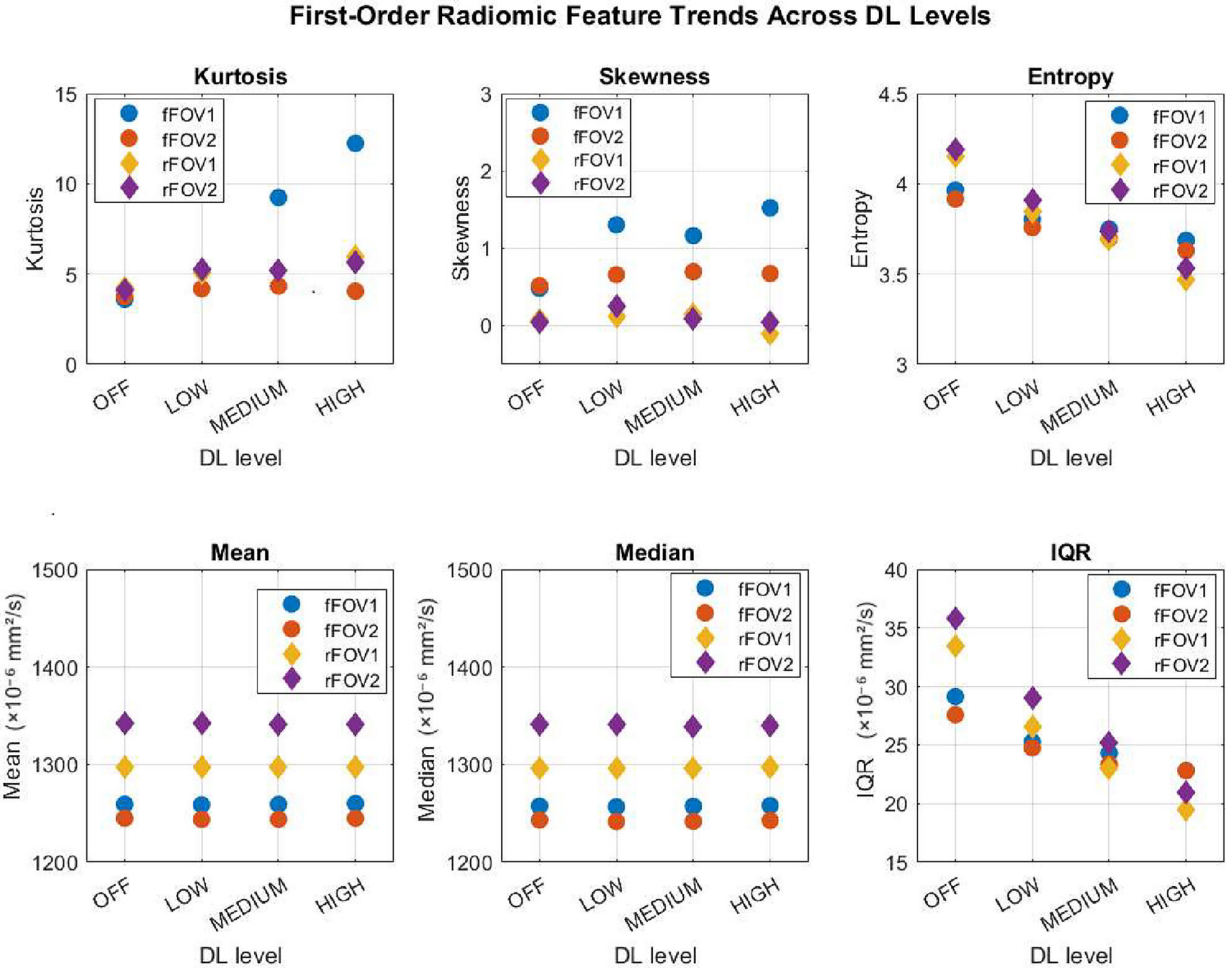
Trends of first-order radiomic features (kurtosis, skewness, entropy, mean, median, and interquartile range or IQR across DL levels for fFOV and rFOV acquisitions in the two repeated sessions

Trends of first-order radiomic features (kurtosis, skewness, entropy, mean, median, and interquartile range or IQR) across DL levels for fFOV and rFOV acquisitions are shown in Fig. 4 (trends of 10th, 25th, 75th, and 90th percentiles are illustrated in Supplementary Fig. S4). The plotted curves represent mean values averaged across all inserts.

**Fig. 5:**
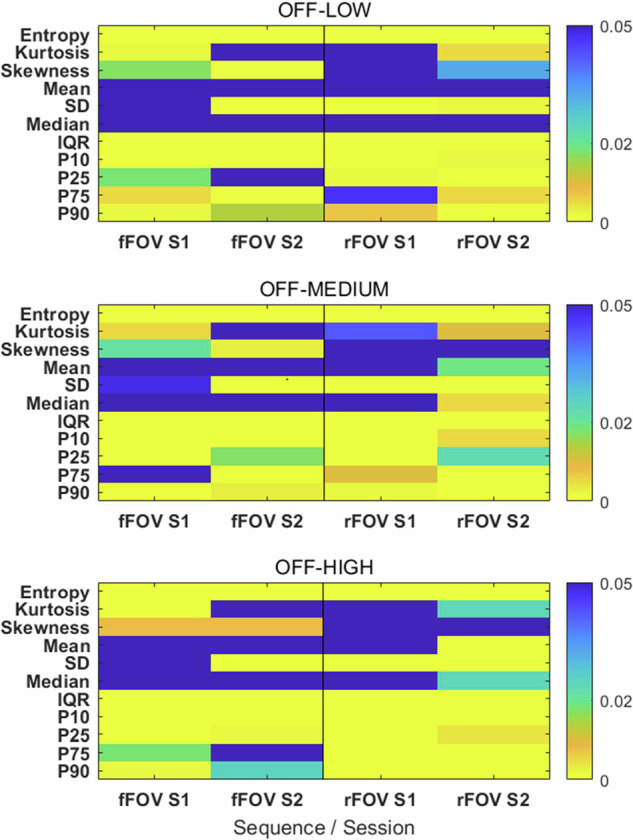
Heatmap of *p*-values from paired Wilcoxon signed-rank tests for ADC first-order radiomic features across DL levels, sequences (fFOV, rFOV), and sessions (S1/S2). Color scaling highlights the significance threshold (*p* ≤ 0.05). SD, Standard deviation; P10, 10th Percentile; P25, 25th Percentile; P75, 75th Percentile; P90, 90th Percentile

Percent differences between DL-OFF and DL-based reconstructions (LOW, MEDIUM, HIGH) for all phantom inserts, aggregated over the two sessions, are reported in Table 4; values are expressed as mean ± standard deviation to summarize the average DL-induced effect across inserts. Session-specific differences are detailed in Supplementary Table S5. Wilcoxon signed-rank test results for individual features comparing the DL OFF condition with the various DL levels are reported in Supplementary Table S6 and illustrated in Fig. 5 as heatmaps, where blue cells indicate non-significant results (*p* ≥ 0.05).

**Table 4:** Percent differences of first-order radiomic features between DL-OFF and DL-based reconstructions (LOW, MEDIUM, HIGH) averaged over all phantom inserts and pooled across sessions

fFOV	Δ(%) OFF_LOW		Δ(%) OFF_MED		Δ(%) OFF_HIGH	
Variable	Mean	SD	Mean	SD	Mean	SD
Entropy	-4.2	± 2.0	-5.5	± 2.0	-7.0	± 2.9
IQR	-10.1	± 6.3	-12.7	± 8.9	-15.9	± 10.8
Kurtosis	89	± 176	70	± 142	103	± 202
Mean	-0.02	± 0.17	-0.01	± 0.15	0.06	± 0.19
Median	-0.04	± 0.18	-0.03	± 0.17	0.04	± 0.22
P10	0.23	± 0.24	0.29	± 0.18	0.44	± 0.31
P25	0.10	± 0.20	0.13	± 0.16	0.25	± 0.26
P75	-0.19	± 0.23	-0.21	± 0.24	-0.19	± 0.27
P90	-0.27	± 0.32	-0.33	± 0.33	-0.33	± 0.37
Skewness	67	± 844	154	± 628	263	± 873
SD	-4.8	± 9.6	-8.8	± 8.8	-9.2	± 15.0

rFOV	Δ(%) OFF_LOW		Δ(%) OFF_MED		Δ(%) OFF_HIGH	
Variable	Mean	SD	Mean	SD	Mean	SD
Entropy	-7.30	± 3.40	-11.08	± 3.62	-16.03	± 4.13
IQR	-19.5	± 6.7	-28.3	± 7.9	-37.6	± 9.5
Kurtosis	21.3	± 24.9	20.9	± 24.2	29.4	± 49.4
Mean	0.07	± 0.21	0.01	± 0.25	-0.07	± 0.12
Median	0.02	± 0.19	-0.03	± 0.21	-0.08	± 0.15
P10	0.60	± 0.52	0.77	± 0.65	0.95	± 0.72
P25	0.33	± 0.28	0.40	± 0.34	0.46	± 0.35
P75	-0.21	± 0.25	-0.38	± 0.29	-0.59	± 0.28
P90	-0.39	± 0.36	-0.65	± 0.47	-1.02	± 0.58
Skewness	41	± 83	46	± 95	31	± 128
SD	-15.9	± 7.6	-23.6	± 10.5	-32.9	± 12.9

*SD* Standard deviation, *P10* 10th Percentile, *P25* 25th Percentile, *P75* 75th Percentile, *P90* 90th Percentile

Entropy and IQR decreased progressively and significantly across all DL levels (*p* < 0.001 for all pairwise comparisons in both fFOV and rFOV), confirming a reduction in ADC heterogeneity with increasing DL strength. The IQR reduction ranged from -10.1% to -15.9% in fFOV and from -19.5% to -37.6% in rFOV acquisitions, while entropy decreased by 4–7% and 7–16%, respectively.

Kurtosis increased across all DL levels, with changes between 70% to 103% in fFOV and from 20.9% to 29.4% in rFOV acquisitions, with greater consistency observed in rFOV acquisitions. Skewness also increased by 67–236% and 31–46%, respectively, although with high variability across inserts and sessions.

Mean and median ADC values remained stable across all DL levels, with percentage differences below 0.3% in all comparisons, despite a few statistically significant *p*-values (notably in the second rFOV session, Fig. 5, and Supplementary Table S6). Lower percentiles (10th and 25th percentiles) showed slight increases (by 0.1–1%). In contrast, the upper percentiles (75th and 90th P75 percentiles) decreased slightly (by 0.1–1%), consistent with the observed reduction in IQR at higher DL levels.

## Discussion

This study evaluated the impact of deep learning–based image reconstruction on ADC quantification and first-order radiomic features in DWI using a calibrated diffusion phantom, as recommended by QIBA to assess quantitative ADC accuracy and reproducibility [17, 19]. By including both conventional full-field-of-view sequences and a reduced-field-of-view sequence [21, 28, 29], we explored whether the influence of DL reconstruction varied across sequence types and spatial resolutions.

Recent studies have investigated the impact of DL reconstruction on DWI across various anatomical regions [15, 30–37], consistently reporting improvements in image quality, SNR, or contrast-to-noise ratio without compromising ADC values, often with reduced acquisition times. Among phantom studies, Lemainque et al [38] used the same commercial diffusion phantom as in the present work, focusing on a comparison between conventional and AI-accelerated (Compressed-Sense) DWI and reporting reduced ADC bias by DL-based reconstruction. While these works predominantly focused on image quality assessments, to our knowledge, no prior studies have explored the impact of DL on the ADC histogram and first-order radiomic features.

In our investigation, DL reconstruction did not introduce systematic bias in ADC quantification, demonstrating that the vendor-provided DL reconstruction model preserved the quantitative accuracy of ADC measurements across sequences and repeated acquisitions [23]. ADC measurements remained stable across DL-based reconstructions, with low CV values and low maximum deviations within each measurement session.

The combined evaluation of ADC histograms, Wasserstein distances, and Friedman test results indicated that DL reconstruction introduced a measurable alteration in ADC histograms. Increasing DL strength led to progressive narrowing of ADC histogram and larger Wasserstein distances, indicating enhanced noise suppression and reduced voxel-level variability. These effects were most evident in the water inserts (PVP0), where high diffusivity and low signal intensity at high *b*-values make ADC estimation more susceptible to smoothing, as previously reported [39]. Conversely, inserts with lower ADC showed minimal changes in the histogram, reflecting their intrinsically greater stability. The effects on ADC histograms were more evident in rFOV acquisitions, likely due to higher spatial resolution and increased sensitivity to DL-driven denoising. Importantly, mean and median ADC values remained stable across all DL levels, with differences below 0.3%, confirming preserved quantitative accuracy. Notably, these ADC differences are well below the variability thresholds defined in the QIBA ADC Profile, which are based on *in vivo* test–retest studies [19]. In fact, detecting true biological changes *in vivo* requires mean ADC changes of approximately 8% in the brain, 15% in the breast, and 27% in the liver and prostate. Therefore, although the clinical relevance of minor changes may depend on anatomy, statistically significant differences observed in our controlled phantom setting are unlikely to be meaningful in most practical applications.

The analysis of first-order radiomic features provided additional insight into the impact of DL reconstruction on ADC map statistics. Significant reductions were found in entropy and the interquartile range, along with convergence of the lower (10th and 25th percentiles) and upper percentiles (75th and 90th percentiles). This indicated that the convolutional neural network effectively suppressed noise and minor ADC fluctuations while preserving the mean water diffusivity.

The observed increase in kurtosis supported this interpretation, considering that more peaked histograms are expected as noise is reduced, with more pronounced effects in rFOV DWI.

The increase in skewness, despite its large variability, led to an alteration in histogram asymmetry without an actual shift in ADC values. The underlying mechanisms are not straightforward and likely reflect interactions between noise properties and DL-driven smoothing, warranting further investigation. The wide percentage differences observed with DL reconstruction should be interpreted in light of the high sensitivity of skewness to distribution tails and its low absolute values in the DL-OFF condition (~ 0.5 for fFOV and 0.05–0.1 for rFOV acquisitions; Fig. 4), such that modest absolute changes in skewness may result in very large relative variations. This behavior also aligns with earlier reproducibility studies, which found that ADC skewness was moderately to poorly stable when acquisition and image pre-processing parameters were varied, compared to the other ADC first-order radiomic features [40, 41].

From a clinical perspective, these findings may be directly relevant to oncologic DWI, where ADC histogram analysis has increasingly been used to quantify tumor heterogeneity, improve tumor grade differentiation, and assess early treatment response [4, 9, 11, 13, 42, 43], being able to capture changes in tissue organization that are not evident from mean ADC values alone. In this context, DL-induced smoothing may enhance the robustness of ADC measurements by reducing noise-related variability; however, it could also attenuate the sensitivity to subtle heterogeneity patterns. This highlights an inherent trade-off between denoising and preservation of fine structural information, which is particularly relevant for radiomic analyses and clinical applications relying on textural heterogeneity.

As an important limitation of the study, we note that it was conducted on a single MRI system equipped with a vendor-specific DL network. As DL implementations may differ in architecture, training data, and integration with the image reconstruction pipeline, the observed effects may be vendor-dependent, and multi-vendor validation studies are required before broader generalization. The limited number of inserts may have reduced the statistical power of the first-order radiomic analysis, leading to some session-to-session variability in the statistical significance of certain features. Moreover, even if the NIST/QIBA phantom is suitable for ADC accuracy and repeatability, it represents a simplified model compared with *in vivo* tissue, lacking realistic microstructural heterogeneity, perfusion effects, and anatomical complexity. Consequently, only first-order radiomic features were evaluated in this study, limiting insight into the impact of DL-based reconstruction on higher-order textural analysis, which captures spatial relationships and finer heterogeneity.

In conclusion, DL-based reconstruction maintained accurate and repeatable ADC quantification, without introducing evident bias. DL effects were instead observed in the shape of the ADC distributions, particularly in the high-ADC regions, resulting in a lower ADC heterogeneity. Clinically, these findings support the use of DL in oncologic DWI, while highlighting the need for further validation in anthropomorphic phantoms and clinical datasets across different tumor types and acquisition protocols.

## Supplementary information

**Additional file 1:** **Fig. S1**, Percentage deviation between measured and nominal ADC values across DL reconstruction levels and acquisition sessions, averaged over all inserts. The solid line represents 0% bias, while dashed lines indicate ±5% bias. **Fig. S2:** Maximum percentage deviation between intra-session measurements for each vial, DL reconstruction level, and type of sequence. **Fig. S3.** Wasserstein distance quantifying the differences in the entire ADC value distribution between OFF and deep learning acquisition levels (LOW, MEDIUM, and HIGH), pairwise, for each insert using fFOV DWI (a) and rFOV DWI (b). **Fig. S4:** Trends of percentile-based first-order radiomic features (10th, 25th, 75th, and 90th percentiles) across deep learning (DL) reconstruction levels for fFOV and rFOV acquisitions in the two repeated sessions. **Table S1.** Estimated fit coefficients and 95% confidence intervals (95% CI) for the ADC–temperature dependence at each PVP concentration. **Table S2.** Session-specific CV values for each insert and DL reconstruction strength. **Table S3** Session-specific Wasserstein distance between OFF and different deep learning acquisition levels (LOW, MEDIUM, and HIGH), pairwise, for each insert using fFOV DWI (a) and rFOV DWI (b). **Table S4.** Friedman test results for the comparison of ADC histograms across DL levels in the two repeated sessions. **Table S5.** Session-specific percent differences of first-order radiomic features, between DL-OFF and DL-based reconstructions (LOW, MEDIUM, HIGH) averaged over all phantom inserts, using fFOV DWI (a) and rFOV DWI (b). **Table S6.** Results of paired Wilcoxon signed-rank test for the comparison of ADC first-order radiomic features across DL levels.

## Data Availability

The datasets used and/or analyzed during the current study are available from the corresponding author on reasonable request.

## Abbreviations

ADC: Apparent diffusion coefficient
CV: Coefficient of variation
DL: Deep learning
DWI: Diffusion-weighted imaging
fFOV: Full field-of-view
IQR: Interquartile range
MRI: Magnetic resonance imaging
NIST: National Institute of Standards and Technology
PVP: Polyvinylpyrrolidone
QIBA: Quantitative Imaging Biomarkers Alliance
rFOV: Reduced field-of-view
SNR: Signal-to-noise ratio
VOI: Volume of interest
