# Supplementary material for: Impact of deep learning image reconstruction on ADC quantification and histogram metrics: a phantom study
Source: Eur Radiol Exp. 2026 Apr 13;10:45. doi: 10.1186/s41747-026-00709-y (PMC13076709; doi:10.1186/s41747-026-00709-y)
Supplement: Supplementary file 1 — Additional file 1: Fig. S1, Percentage deviation between measured and nominal ADC values across DL reconstruction levels and acquisition sessions, averaged over all inserts. The solid line represents 0% bias, while dashed lines indicate ±5% bias. Fig. S2: Maximum percentage deviation between intra-session measurements for each vial, DL reconstruction level, and type of sequence. Fig. S3. Wasserstein distance quantifying the differences in the entire ADC value distribution between OFF and deep learning acquisition levels (LOW, MEDIUM, and HIGH), pairwise, for each insert using fFOV DWI (a) and rFOV DWI (b). Fig. S4: Trends of percentile-based first-order radiomic features (10th, 25th, 75th, and 90th percentiles) across deep learning (DL) reconstruction levels for fFOV and rFOV acquisitions in the two repeated sessions. Table S1. Estimated fit coefficients and 95% confidence intervals (95% CI) for the ADC–temperature dependence at each PVP concentration. Table S2. Session-specific CV values for each insert and DL reconstruction strength. Table S3 Session-specific Wasserstein distance between OFF and different deep learning acquisition levels (LOW, MEDIUM, and HIGH), pairwise, for each insert using fFOV DWI (a) and rFOV DWI (b). Table S4. Friedman test results for the comparison of ADC histograms across DL levels in the two repeated sessions. Table S5. Session-specific percent differences of first-order radiomic features, between DL-OFF and DL-based reconstructions (LOW, MEDIUM, HIGH) averaged over all phantom inserts, using fFOV DWI (a) and rFOV DWI (b). Table S6. Results of paired Wilcoxon signed-rank test for the comparison of ADC first-order radiomic features across DL levels. [file 41747_2026_709_MOESM1_ESM.pdf]

Impact of deep learning image reconstruction on ADC quantification and histogram metrics: a phantom study

ELECTRONIC SUPPLEMENTARY MATERIAL

Supplementary Figures

Supplementary Figure S1. Percentage deviation between measured and nominal ADC values across DL reconstruction levels and acquisition sessions, averaged over all inserts. The solid line represents 0% bias, while dashed lines indicate ±5% bias.

Supplementary Figure S2: Maximum percentage deviation between intra-session measurements for each vial, DL reconstruction level, and type of sequence

Supplementary Figure S3. Wasserstein distance quantifying the differences in the entire ADC value distribution between OFF and deep learning acquisition levels (LOW, MEDIUM, and HIGH), pairwise, for each insert using fFOV DWI (a) and rFOV DWI (b).**Supplementary Figure S4:** Trends of percentile-based first-order radiomic features (10th, 25th, 75th, and 90th percentiles) across deep learning (DL) reconstruction levels for fFOV and rFOV acquisitions in the two repeated sessions.

Supplementary Tables

Supplementary Table S1. Estimated fit coefficients and 95% confidence intervals (95% CI) for the ADC–temperature dependence at each PVP concentration

Supplementary Table S2. Session-specific CV values for each insert and DL reconstruction strength

Supplementary Table S3 Session-specific Wasserstein distance between OFF and different deep learning acquisition levels (LOW, MEDIUM, and HIGH), pairwise, for each insert using fFOV DWI (a) and rFOV DWI (b)

Supplementary Table S4. Friedman test results for the comparison of ADC histograms across DL levels in the two repeated sessions

Supplementary Table S5. Session-specific percent differences of first-order radiomic features, between DL-OFF and DL-based reconstructions (LOW, MEDIUM, HIGH) averaged over all phantom inserts, using fFOV DWI (a) and rFOV DWI (b)

Supplementary Table S6. Results of paired Wilcoxon signed-rank test for the comparison of ADC first-order radiomic features across DL levels

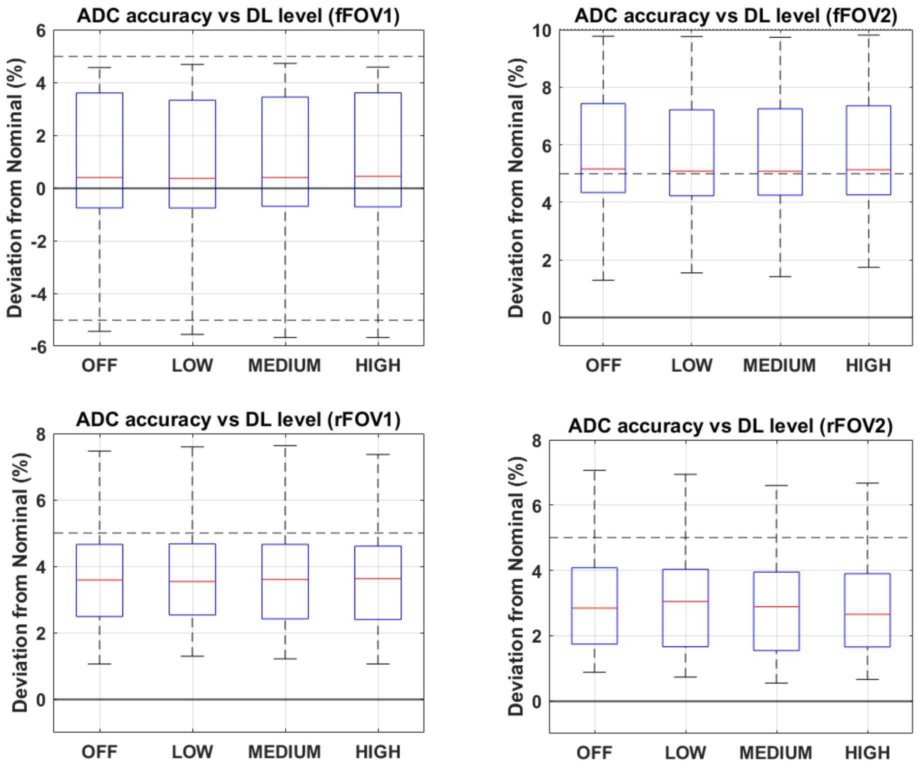

Supplementary Figure S1. Percentage deviation between measured and nominal ADC values across DL reconstruction levels and acquisition sessions, averaged over all inserts. The solid line represents 0% bias, while dashed lines indicate ±5% bias.

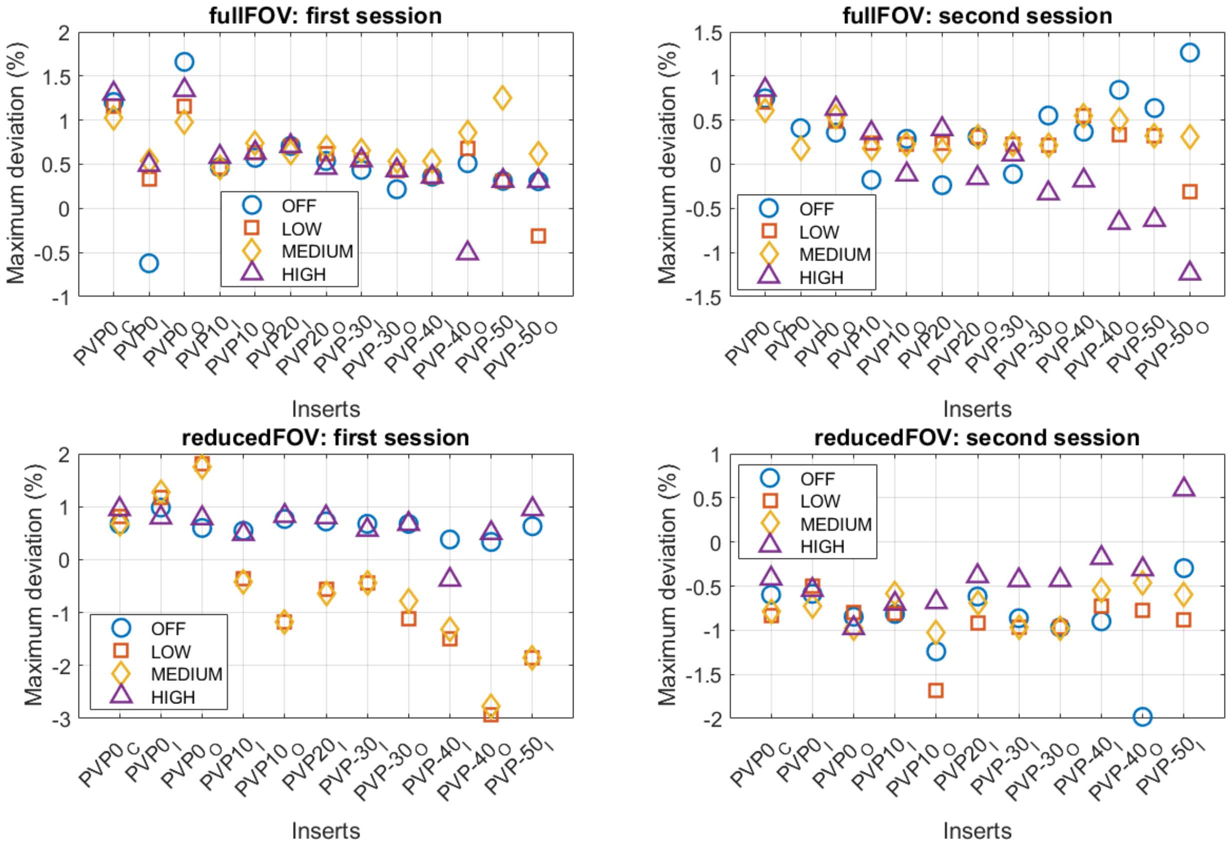

Supplementary Figure S2: Maximum percentage deviation between intra-session measurements for each vial, DL reconstruction level, and type of sequence.

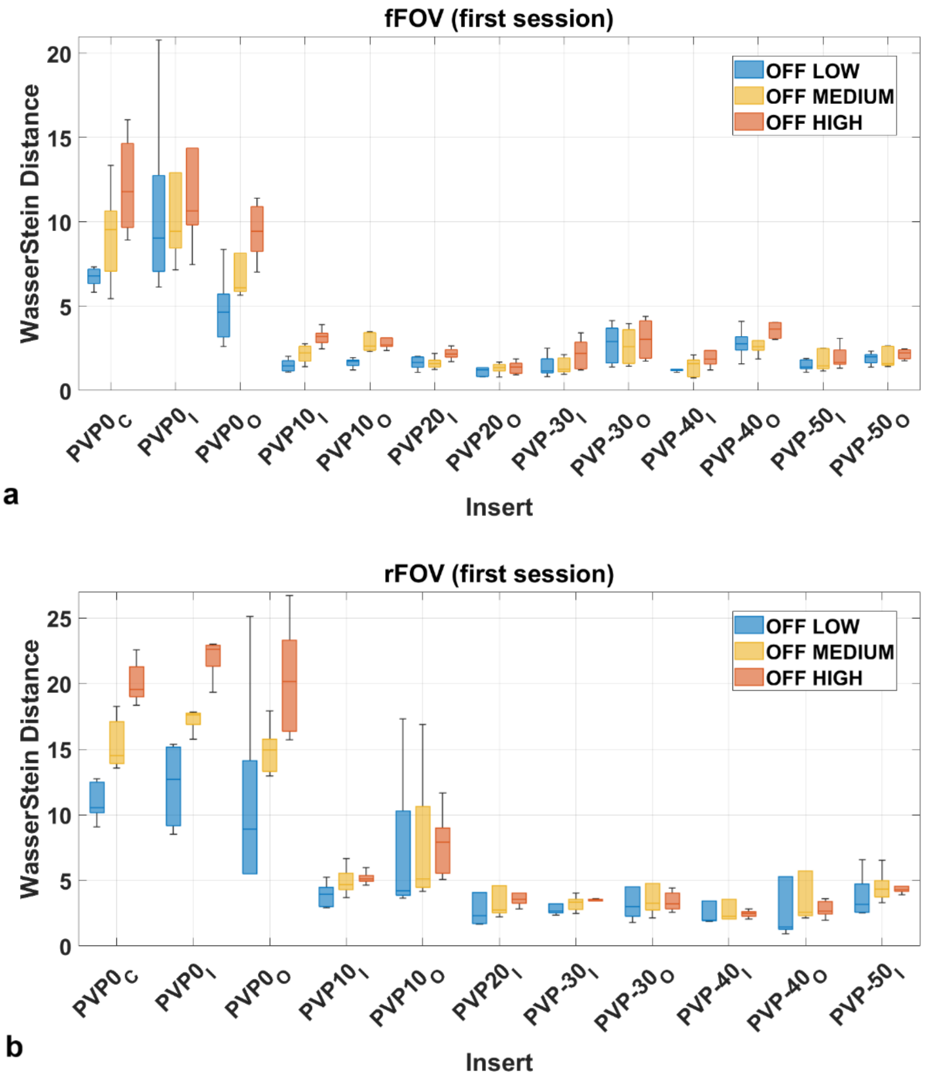

Supplementary Figure S3. Wasserstein distance quantifying the differences in the entire ADC value distribution between OFF and deep learning acquisition levels (LOW, MEDIUM, and HIGH), pairwise, for each insert using fFOV DWI (a) and rFOV DWI (b). Comparable trends were observed in the plots during the second measurement session.

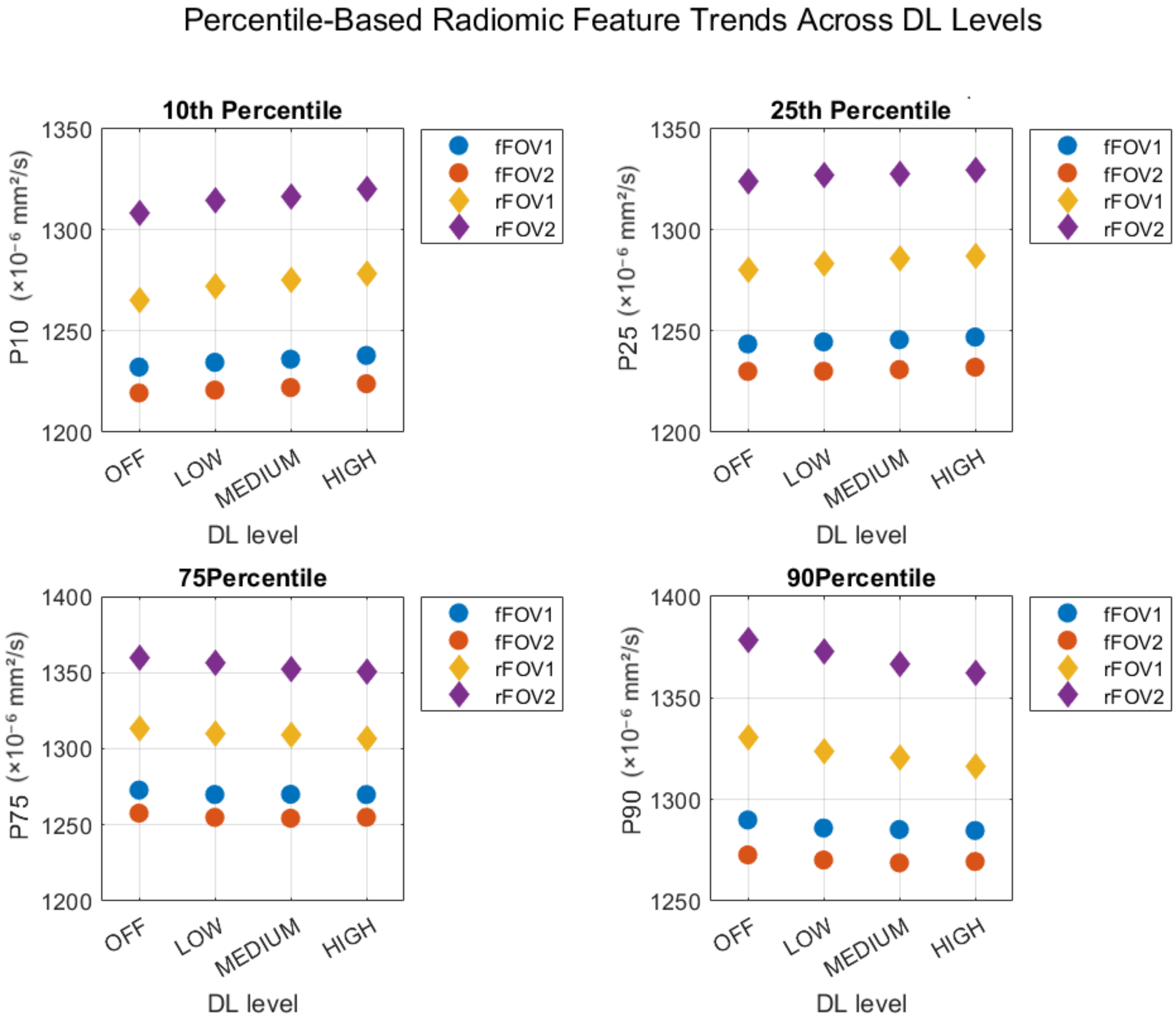

Supplementary Figure S4: Trends of percentile-based first-order radiomic features (10th, 25th, 75th, and 90th percentiles) averaged over all inserts, across deep learning (DL) reconstruction levels for fFOV and rFOV acquisitions in the two repeated sessions.

Supplementary Table S1. Estimated fit coefficients and 95% confidence intervals for the ADC–temperature dependence derived from NIST and CaliberMRI reference data at each PVP concentration

PVP	a	b	c	R2
0% (H_2O)	0.40<br>[0.35, 0.44]	36.1<br>[34.4, 37.9]	1130<br>[1113, 1146]	1.000
10%	0.39<br>[0.36, 0.42]	28.0<br>[26.9, 29.0]	831<br>[821, 841]	1.000
20%	0.30<br>[0.28, 0.32]	22.8<br>[22.0, 23.6]	594<br>[587, 602]	1.000
30%	0.28<br>[0.25, 0.30]	16.6<br>[15.6, 17.6]	403<br>[393, 413]	1.000
40%	0.23<br>[0.21, 0.26]	9.8<br>[8.8, 10.9]	214<br>[204, 224]	1.000
50%	0.19<br>[0.16, 0.23]	5.3<br>[4.0, 6.7]	117<br>[103, 130]	1.000

Abbreviations: CI, confidence interval; R^2, coefficient of determination. The fit model was defined as: ADC (10^{-6} mm^2/s) = $a*T(°C)^2 + b*T(°C) + c$;

Supplementary Table S2. Session-specific CV values (%) for each insert and DL reconstruction strength

(a)

fFOV (1th session)				
**insert**	**OFF**	**LOW**	**MEDIUM**	**HIGH**
PVP0_C	0.46	0.48	0.46	0.51
PVP0_I	0.26	0.13	0.24	0.21
PVP0_O	0.65	0.48	0.48	0.56
PVP10_I	0.20	0.20	0.20	0.25
PVP10_O	0.24	0.27	0.29	0.23
PVP20_I	0.28	0.28	0.25	0.27
PVP20_O	0.23	0.27	0.28	0.20
PVP30_I	0.16	0.20	0.24	0.21
PVP30_O	0.09	0.17	0.20	0.17
PVP40_I	0.20	0.15	0.20	0.20
PVP40_O	0.27	0.28	0.33	0.30
PVP50_I	0.17	0.22	0.47	0.17
PVP50_O	0.17	0.14	0.28	0.17
**fFOV (2nd session)**				
**insert**	**OFF**	**LOW**	**MEDIUM**	**HIGH**
PVP0_C	0.33	0.26	0.22	0.34
PVP0_I	0.15	0.19	0.10	0.32
PVP0_O	0.20	0.22	0.21	0.29
PVP10_I	0.09	0.09	0.11	0.13
PVP10_O	0.15	0.12	0.12	0.09
PVP20_I	0.09	0.10	0.11	0.15
PVP20_O	0.14	0.12	0.12	0.12
PVP30_I	0.11	0.10	0.09	0.06
PVP30_O	0.21	0.13	0.11	0.13
PVP40_I	0.15	0.20	0.20	0.10
PVP40_O	0.35	0.22	0.22	0.29
PVP50_I	0.32	0.14	0.17	0.28
PVP50_O	0.48	0.22	0.17	0.51

(b)

rFOV (1th session)				
**insert**	**OFF**	**LOW**	**MEDIUM**	**HIGH**
PVP0_C	0.32	0.37	0.31	0.37
PVP0_I	0.37	0.49	0.50	0.33
PVP0_O	0.22	0.73	0.67	0.37
PVP10_I	0.23	0.17	0.23	0.22
PVP10_O	0.35	0.46	0.45	0.38
PVP20_I	0.29	0.27	0.31	0.34
PVP-30_I	0.27	0.23	0.29	0.25
PVP-30_O	0.30	0.44	0.33	0.35
PVP-40_I	0.17	0.59	0.54	0.22
PVP-40_O	0.20	1.24	1.13	0.31
PVP-50_I	0.41	0.74	0.78	0.41
**rFOV (2nd session)**				
**insert**	**OFF**	**LOW**	**MEDIUM**	**HIGH**
PVP0_C	0.28	0.32	0.32	0.16
PVP0_I	0.24	0.36	0.31	0.20
PVP0_O	0.45	0.36	0.36	0.41
PVP10_I	0.30	0.32	0.27	0.28
PVP10_O	0.50	0.66	0.42	0.32
PVP20_I	0.23	0.38	0.26	0.15
PVP-30_I	0.32	0.39	0.36	0.19
PVP-30_O	0.39	0.41	0.35	0.16
PVP-40_I	0.35	0.30	0.24	0.10
PVP-40_O	0.77	0.30	0.33	0.17
PVP-50_I	0.21	0.39	0.45	0.39

Supplementary Table S3 Session-specific Wasserstein distance between OFF and different deep learning acquisition levels (LOW, MEDIUM, and HIGH), pairwise, for each insert using fFOV (a) and rFOV DWI (b)

(a)

**fFOV (1th session)**	**OFF-LOW**		**OFF-MEDIUM**		**OFF-HIGH**	
**Insert**	**median**	**IQR**	**median**	**IQR**	**median**	**IQR**
PVP0_C	6.8	0.9	9.5	3.6	11.8	5.0
PVP0_I	9.0	5.7	9.5	4.5	10.7	4.6
PVP0_O	4.6	2.5	6.1	2.3	9.4	2.7
PVP10_I	1.5	0.6	2.2	0.9	3.2	0.6
PVP10_O	1.7	0.3	2.6	1.0	2.7	0.5
PVP20_I	1.6	0.6	1.6	0.4	2.1	0.5
PVP20_O	1.2	0.5	1.3	0.4	1.4	0.6
PVP-30_I	1.2	0.8	1.3	0.8	2.2	1.6
PVP-30_O	2.9	2.1	2.6	2.0	3.0	2.2
PVP-40_I	1.2	0.1	1.6	1.0	1.9	0.8
PVP-40_O	2.8	0.8	2.6	0.6	3.7	0.9
PVP-50_I	1.4	0.5	1.5	1.2	1.7	0.8
PVP-50_O	2.0	0.5	1.6	1.1	2.2	0.5
**fFOV (2nd session)**	**OFF-LOW**		**OFF-MEDIUM**		**OFF-HIGH**	
**insert**	**median**	**IQR**	**median**	**IQR**	**median**	**IQR**
PVP0_C	8.5	3.8	9.5	2.6	11.8	1.6
PVP0_I	8.1	2.8	7.8	0.9	8.7	5.5
PVP0_O	3.7	0.8	6.0	2.0	7.1	1.1
PVP10_I	2.0	1.4	2.0	2.2	3.0	1.7
PVP10_O	1.3	0.3	1.7	0.3	2.5	1.2
PVP20_I	1.1	1.6	1.8	1.2	1.9	2.0
PVP20_O	1.0	0.5	1.0	0.3	1.5	1.1
PVP-30_I	1.4	0.4	1.2	0.4	1.7	0.7
PVP-30_O	1.7	1.0	1.7	0.4	1.9	1.8
PVP-40_I	0.8	0.2	0.8	0.4	1.2	0.8
PVP-40_O	1.9	0.7	2.2	0.6	2.5	2.1
PVP-50_I	1.4	0.6	1.4	0.1	2.0	1.2
PVP-50_O	1.8	0.5	1.9	0.3	2.0	1.4

(b)

rFOV (1th session)	OFF-LOW		OFF-MEDIUM		OFF-HIGH	
**Insert**	**median**	**IQR**	**median**	**IQR**	**median**	**IQR**
PVP0_C	10.5	2.3	14.5	3.2	19.6	2.3
PVP0_I	12.7	6.0	17.6	0.9	22.6	1.6
PVP0_O	8.9	8.7	15.0	2.5	20.1	6.9
PVP10_I	4.0	1.5	4.7	1.2	5.1	0.5
PVP10_O	4.2	6.4	5.1	6.2	7.9	3.4
PVP20_I	2.3	2.4	2.8	2.1	3.6	0.8
PVP-30_I	2.6	0.7	3.4	0.8	3.5	0.1
PVP-30_O	3.0	2.3	3.3	2.0	3.2	1.2
PVP-40_I	1.9	1.5	2.3	1.5	2.5	0.4
PVP-40_O	1.4	4.0	2.6	3.4	2.7	0.9
PVP-50_I	3.2	2.1	4.3	1.2	4.3	0.4
**rFOV (2nd session)**	**OFF-LOW**		**OFF-MEDIUM**		**OFF-HIGH**	
Insert	median	IQR	median	IQR	median	IQR
PVP0_C	12.6	6.6	18.3	4.7	23.8	1.9
PVP0_I	9.9	1.8	16.1	3.3	19.6	6.7
PVP0_O	6.4	1.6	15.2	5.1	22.3	7.2
PVP10_I	3.9	2.0	5.8	1.8	7.2	2.0
PVP10_O	3.9	1.3	9.0	7.0	10.4	4.8
PVP20_I	3.6	1.9	3.5	1.4	4.1	1.2
PVP-30_I	2.3	0.8	3.3	1.2	3.4	0.3
PVP-30_O	2.7	0.8	4.1	3.5	3.8	1.5
PVP-40_I	2.0	0.3	2.3	1.0	2.5	0.4
PVP-40_O	2.0	1.7	3.3	3.3	3.8	3.8
PVP-50_I	3.0	0.6	3.3	0.9	4.9	0.3

Data are expressed as median and interquartile range (IQR).

Supplementary Table S4. Friedman test results for the comparison of ADC histograms across DL levels in the two repeated sessions

Friedman test	fFOV		rFOV	
	1th session	2nd session	1th session	2nd session
Insert	p-value	p-value	p-value	p-value
PVP0_C	**<0.001**	**<0.001**	**<0.001**	**<0.001**
PVP0_I	**<0.001**	**<0.001**	**<0.001**	**<0.001**
PVP0_O	**<0.001**	**<0.001**	**<0.001**	**<0.001**
PVP10_I	**0.030**	**0.047**	**0.001**	**<0.001**
PVP10_O	0.017	0.191	**<0.001**	**<0.001**
PVP20_I	0.527	0.004	**0.006**	**0.007**
PVP20_O	0.215	0.288	-	-
PVP-30_I	**<0.001**	**0.025**	**0.001**	**0.008**
PVP-30_O	0.004	0.625	0.005	0.054
PVP-40_I	0.630	0.043	**<0.001**	**0.008**
PVP-40_O	0.003	0.929	0.300	<0.001
PVP-50_I	0.161	0.288	**0.020**	**<0.001**
PVP-50_O	0.286	0.005	-	-

Abbreviations: PVP, polyvinylpyrrolidone solutions of six concentrations (0%, 10%, 20%, 30%, 40%, and 50% by weight), arranged in the middle (C), inner (I), and outer (O) rings.
The *p* values that reached significance in both measurement sessions are in **bold**.

Supplementary Table S5. Session-specific percent differences of first-order radiomic features, between DL-OFF and DL-based reconstructions (LOW, MEDIUM, HIGH) averaged over all phantom inserts, using fFOV DWI (a) and rFOV DWI (b)

(a)

**fFOV** 1th session	D(%) OFF_LOW		D(%) OFF_MED		D(%) OFF_HIGH	
Variable	Mean	SD	Mean	SD	Mean	SD
Entropy	-4.2	2.2	-5.4	2.3	-6.8	3.5
IQR	-11.3	7.0	-12.9	9.9	-17.5	12.7
Kurtosis	174	219	132	182	205	248
Mean	-0.01	0.18	0.01	0.18	0.05	0.17
Median	-0.03	0.16	-0.01	0.18	0.01	0.17
P10	0.25	0.18	0.34	0.18	0.41	0.23
P25	0.10	0.16	0.15	0.16	0.21	0.20
P75	-0.21	0.28	-0.18	0.28	-0.23	0.31
P90	-0.34	0.36	-0.38	0.36	-0.43	0.42
Skewness	256	1064	250	889	335	1097
SD	-1.3	12.1	-6.3	11.2	-3.9	19.4
**fFOV** 2nd session	D(%) OFF_LOW		D(%) OFF_MED		D(%) OFF_HIGH	
Variable	Mean	SD	Mean	SD	Mean	SD
Entropy	-4.2	1.8	-5.6	1.9	-7.3	2.4
IQR	-9.0	5.6	-12.4	8.1	-14.2	8.7
Kurtosis	3	21	7	24	0	20
Mean	-0.03	0.16	-0.04	0.12	0.08	0.22
Median	-0.04	0.20	-0.05	0.17	0.07	0.27
P10	0.21	0.29	0.25	0.18	0.47	0.37
P25	0.10	0.23	0.12	0.16	0.28	0.32
P75	-0.18	0.18	-0.23	0.21	-0.14	0.24
P90	-0.20	0.26	-0.29	0.30	-0.23	0.30
Skewness	-123	523	58	112	191	612
SD	-8.4	4.1	-11.3	4.8	-14.6	5.4

(b)

**rFOV** 1th session	D(%) OFF_LOW		D(%) OFF_MED		D(%) OFF_HIGH	
Variable	Mean	SD	Mean	SD	Mean	SD
Entropy	-7.5	2.9	-11.1	3.3	-16.3	4.2
IQR	-19.6	6.8	-27.7	9.0	-36.7	11.2
Kurtosis	17	27	18	25	30	61
Mean	0.09	0.23	0.12	0.23	0.00	0.08
Median	0.06	0.21	0.08	0.20	0.00	0.14
P10	0.66	0.53	0.91	0.58	1.05	0.68
P25	0.38	0.31	0.52	0.31	0.53	0.35
P75	-0.19	0.29	-0.28	0.33	-0.50	0.27
P90	-0.41	0.42	-0.59	0.52	-0.99	0.59
Skewness	50	81	56	98	46	141
SD	-17.0	6.5	-24.8	9.3	-34.0	12.7
**rFOV** 2nd session	D(%) OFF_LOW		D(%) OFF_MED		D(%) OFF_HIGH	
Variable	Mean	SD	Mean	SD	Mean	SD
Entropy	-7.1	4.0	-11.0	4.1	-15.8	4.3
IQR	-19.3	6.9	-28.8	7.0	-38.5	8.0
Kurtosis	26	22	23	24	29	37
Mean	0.05	0.20	-0.10	0.22	-0.15	0.11
Median	-0.01	0.18	-0.14	0.17	-0.15	0.12
P10	0.53	0.53	0.62	0.70	0.86	0.77
P25	0.28	0.25	0.28	0.33	0.38	0.35
P75	-0.23	0.22	-0.49	0.20	-0.68	0.27
P90	-0.36	0.32	-0.71	0.42	-1.05	0.60
Skewness	33	87	36	96	16	120
SD	-14.7	8.6	-22.3	11.8	-31.8	13.7

Abbreviations: SD, standard deviation; IQR, interquartile range

Supplementary Table S6. Results of paired Wilcoxon signed-rank test for the comparison of ADC first-order radiomic features across DL levels

Variable	DL levels (Pair)	fFOV		rFOV	
		1th session	2nd session	1th session	2nd session
		p-value	*p-value*	*p-value*	*p-value*
Entropy	OFF-LOW	**<0.001**	**<0.001**	**0.001**	**0.001**
	OFF-MEDIUM	**<0.001**	**<0.001**	**0.001**	**0.001**
	OFF-HIGH	**<0.001**	**<0.001**	**0.001**	**0.001**
Kurtosis	OFF-LOW	0.002	0.946	0.067	0.005
	OFF-MEDIUM	0.005	1.000	**0.042**	**0.010**
	OFF-HIGH	0.001	0.340	0.123	0.024
Skewness	OFF-LOW	**0.017**	**0.001**	0.700	0.032
	OFF-MEDIUM	**0.021**	**0.003**	0.638	0.520
	OFF-HIGH	**0.008**	**0.008**	0.520	1.000
Mean	OFF-LOW	0.839	0.305	0.365	0.577
	OFF-MEDIUM	0.542	0.191	0.638	0.019
	OFF-HIGH	0.244	0.839	0.520	0.001
SD	OFF-LOW	0.216	<0.001	**0.001**	**0.002**
	OFF-MEDIUM	**0.048**	**<0.001**	**0.001**	**0.001**
	OFF-HIGH	0.191	<0.001	**0.001**	**0.001**
Median	OFF-LOW	0.554	0.132	0.449	0.533
	OFF-MEDIUM	0.964	0.097	0.484	0.005
	OFF-HIGH	0.825	0.777	0.715	0.024
IQR	OFF-LOW	**<0.001**	**<0.001**	**0.001**	**0.001**
	OFF-MEDIUM	**<0.001**	**<0.001**	**0.001**	**0.001**
	OFF-HIGH	**<0.001**	**<0.001**	**0.001**	**0.001**
P10	OFF-LOW	**0.001**	**0.001**	**0.001**	**0.002**
	OFF-MEDIUM	**<0.001**	**<0.001**	**0.001**	**0.005**
	OFF-HIGH	**<0.001**	**<0.001**	**0.001**	**0.001**
P25	OFF-LOW	0.018	0.690	**0.002**	**0.001**
	OFF-MEDIUM	**0.001**	**0.017**	**0.001**	**0.023**
	OFF-HIGH	**0.001**	**0.002**	**0.001**	**0.004**
P75	OFF-LOW	**0.005**	**0.001**	**0.047**	**0.005**
	OFF-MEDIUM	0.085	<0.001	**0.010**	**0.001**
	OFF-HIGH	0.018	0.094	**0.001**	**0.001**
P90	OFF-LOW	**0.002**	**0.014**	**0.007**	**0.001**
	OFF-MEDIUM	**<0.001**	**0.003**	**0.002**	**0.001**
	OFF-HIGH	**0.002**	**0.025**	**0.001**	**0.001**

The *p* values that reached significance in both measurement sessions are in **bold**.
